# Air-stable, recyclable, and time-efficient diphenylphosphinite cellulose-supported palladium nanoparticles as a catalyst for Suzuki–Miyaura reactions

**DOI:** 10.3762/bjoc.7.48

**Published:** 2011-03-30

**Authors:** Qingwei Du, Yiqun Li

**Affiliations:** 1 Department of Chemistry, Jinan University, 510632 Guangzhou, China

**Keywords:** diphenylphosphinite cellulose, heterogeneous catalysis, nanopalladium, polymer-supported catalyst, Suzuki–Miyaura reaction

## Abstract

A diphenylphosphinite cellulose palladium complex (Cell–OPPh_2_–Pd^0^) was found to be a highly efficient heterogeneous catalyst for the Suzuki–Miyaura reaction. The products were obtained in good to excellent yield under mild reaction conditions. Moreover, the catalyst could be easily recovered by simple filtration and reused for at least 6 cycles without losing its activity.

## Introduction

The formation of C_sp2_–C_sp2_ bonds has long remained a difficult task until the development of the Suzuki–Miyaura palladium-catalyzed reaction [1–3]. The palladium-catalyzed Suzuki cross-coupling reaction of aryl halides with arylboronic acids is one of the most powerful tools for the preparation of unsymmetrical biaryl compounds [4] and has been applied to many areas, including pharmaceuticals, herbicides, and natural products, as well as in the fields of engineering materials, such as conducting polymers, molecular wires, liquid crystals and synthesis of ligands [5–6]. In the past few years, many efficient and selective catalytic systems have been developed for the reaction. A considerable number of homogenous palladium catalysts have been used to obtain high yields of a desired product [7–9], for example, phosphorus ligands [10–12], N-heterocyclic carbenes [13–15], P,O-based ligands [16], bis(thiourea) ligands [17], and thiosemicarbazone [18], etc. However, the separation of the catalyst and ligands from the final product is problematic. In this regard, studies on heterogeneous catalysts have drawn much attention because they can be easily separated and recovered [19–22]. Recently, numerous solid-supported palladium catalysts have been reported which can be used under mild and/or environmentally benign reaction conditions. These supported catalysts were prepared by immobilizing palladium(II) on supported ligands [23–25] or palladium(0) nanoparticles on various solid supports [26–31] (polystyrene [26], silica [27], cellulose [28], corn starch [29], polymethyl methacrylate [30] and others [31]). In addition, cellulose as efficient support for Pd nanoparticles in other cross-coupling and related reactions is also widely used [32–34].

Herein, we report the synthesis of a novel diphenylphosphinite cellulose palladium complex (Cell–OPPh_2_–Pd^0^) by a simple procedure from diphenylphosphinite funtionalized cellulose (Cell–OPPh_2_) and PdCl_2_ in ethanol solution [35–44]. This class of supported palladium catalysts would solve the basic problems of homogeneous catalysts, i.e., the separation and recycling of the catalysts. This palladium complex catalyst also has the advantage of avoiding contamination of the products by residue ligand and metal. Moreover, it showed excellent catalytic activity in the Suzuki–Miyaura coupling reaction of various aryl haildes bearing electron-withdrawing and/or electron-donating groups.

## Results and Discussion

### Synthesis and characterization of Cell–OPPh_2_ and Cell–OPPh_2_–Pd^0^

**Scheme 1:**
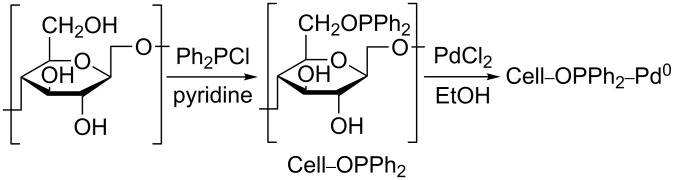
Preparation of the Cell–OPPh_2_–Pd^0^.

It is well known that nanopalladium shows unique reactivity in various organic reactions. However, it is very difficult to use the nanopalladium as practical catalyst because of its tendency to agglomerate and its sensitivity to air and moisture. The Cell–OPPh_2_–Pd^0^ catalyst was prepared with diphenylphosphinite cellulose and palladium dichloride in ethanol (Scheme 1). The as-prepared Cell–OPPh_2_–Pd^0^ catalyst was characterized by inductively coupled plasma-atomic emission spectrometry (ICP-AES), Fourier transform infrared spectroscopy (FTIR), X-ray diffraction (XRD), thermogravimetric analyses (TGA), scanning electron microscopy (SEM) and transmission electron microscopy (TEM).

**Figure 1:**
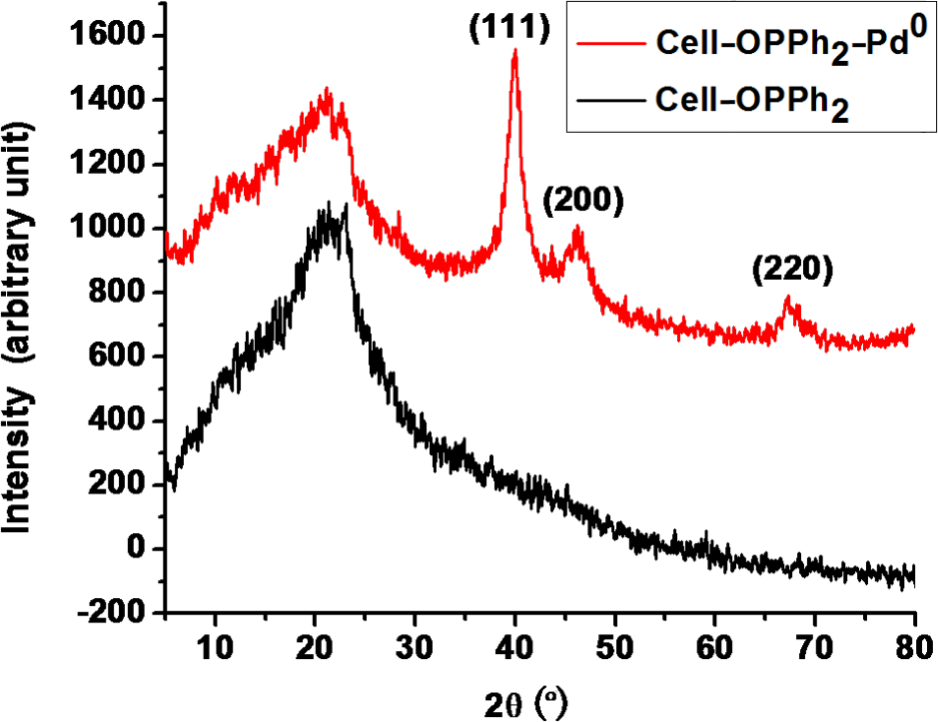
XRD pattern of the cellulose-supported palladium catalyst and Cell–OPPh_2_ matrix.

The Pd loading was determined to be 0.33 mmol/g by ICP-AES. The IR spectra of Cell–OPPh_2_ and Cell–OPPh_2_–Pd^0^ catalysts were recorded. The IR spectrum of Cell–OPPh_2_ contained absorption bands at 1029.9 cm^−1^ for C–O–P bond. This band is negatively shifted to 1028.8 cm^−1^ in the Cell–OPPh_2_–Pd^0^, indicating coordination of the phosphine atom with the palladium and further confirming the formation of a palladium complex on the surface of the polymer. Figure 1 shows the XRD pattern of the cellulose-supported palladium catalyst and the Cell–OPPh_2_ matrix. The Cell–OPPh_2_ displays a broad diffraction with 2θ ranging from 5° to 35°, suggesting the amorphous structure of the polymeric scaffold. Besides the broad diffraction ascribed to the polymeric scaffold, other three diffraction peaks at 2θ of 40.0°, 46.1°, and 67.5° for the diffraction of the (111), (200) and (220) lattice planes of the face-centered cubic crystalline structure of the Pd nanoparticles are correspondingly clearly seen from the XRD pattern of Cell–OPPh_2_–Pd^0^ catalyst.

**Figure 2:**
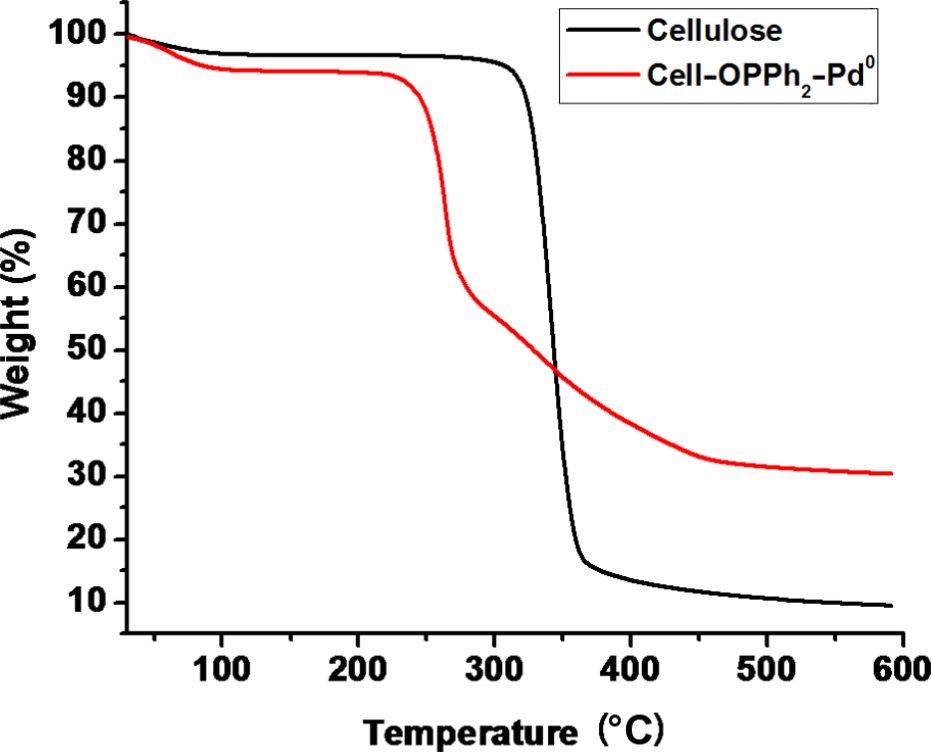
TG curve of the cellulose and Cell–OPPh_2_–Pd^0^ under nitrogen flow.

The thermal stability of Cell–OPPh_2_–Pd^0^ has a great effect on its catalytic activity and recyclability because the Suzuki–Miyaura reaction usually requires heating. TGA of the catalyst systems demonstrated high thermal stability with decomposition starting at around 250 °C under a nitrogen atmosphere. An initial weight loss of around 2.5% was observed up to 100 °C, likely due to the release of adsorbed water (Figure 2).

**Figure 3:**
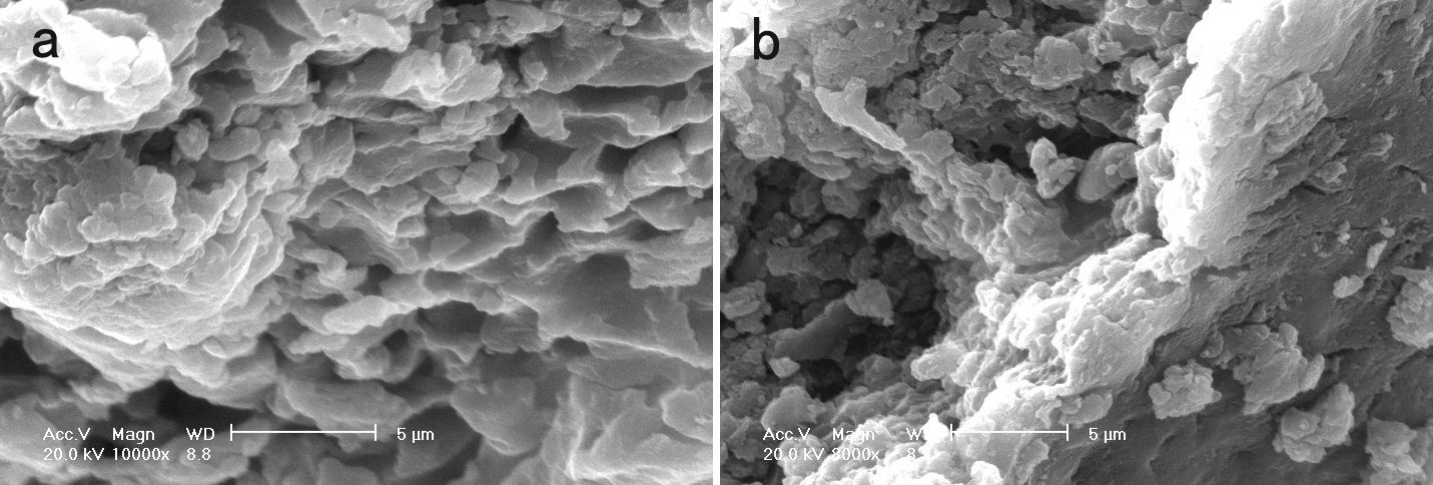
SEM images of the Cell–OPPh_2_ (a) and the fresh catalyst Cell–OPPh_2_–Pd^0^ (b).

**Figure 4:**
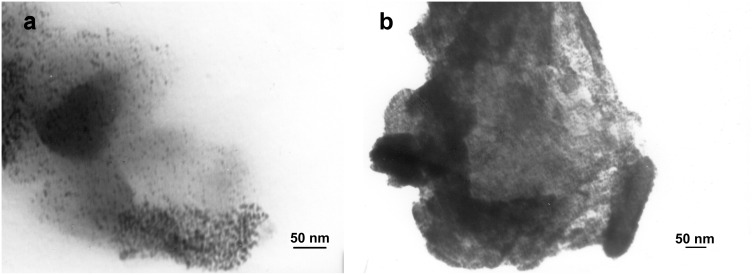
TEM image of the fresh Cell–OPPh_2_–Pd^0^ catalyst (a) and the recovered catalyst after being reused six times (b) at the same magnification.

The morphology of Cell–OPPh_2_–Pd^0^ and Cell–OPPh_2_ was studied by SEM and TEM. A clear change in morphology is observed after anchoring palladium onto the polymer support (Figure 3). The TEM image of the Cell–OPPh_2_–Pd^0^ catalyst shows that the average size of the nanopalladium particles is in the range of 4–15 nm (Figure 4a). The TEM image of the used catalyst indicates that the size and morphology of the nanopalladium has suffered slightly from agglomeration in the recovered catalyst after being reused six times (Figure 4b).

### Suzuki–Miyaura cross-coupling reactions

**Scheme 2:**
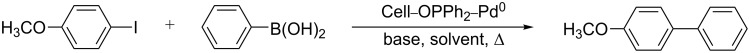
Reaction of 4-iodoanisole with phenylboronic acid.

To explore the efficiency of the diphenylphosphinite cellulose-supported nanopalladium catalyst, it was initially used in the Suzuki–Miyaura cross-coupling reaction, which is a versatile and a well studied method for the generation of C_sp2_–C_sp2_ bonds in organic synthesis. The influence of base, solvent and amount of catalyst on Suzuki–Miyaura cross-coupling were carefully examined with the reaction of 4-iodoanisole and phenylboronic acid chosen as the model reaction (Scheme 2). The results are summarized in Table 1, Table 2 and Table 3, respectively.

**Table 1:** Effect of base on the Suzuki–Miyaura cross-coupling reaction^a^.

Entry	Base	Time (min)	Yield^b^ (%)

1	K_2_CO_3_	15	93
2	Na_2_CO_3_	300	40
3	Cs_2_CO_3_	35	75
4	NaOH	25	70
5	K_3_PO_4_·3H_2_O	20	78
6	CH_3_ONa	14	85
7	KOH	60	40
8	Na_3_PO_4_·12H_2_O	120	85

^a^Reaction conditions: 4-iodoanisole (1.0 mmol), phenylboronic acid (1.2 mmol), base (2.0 mmol), Cell–OPPh_2_–Pd^0^ (0.015 g, 0.005 mmol of Pd), and 5.0 cm^3^ 95% ethanol heating under reflux in air. ^b^Isolated yield based on 4-iodoanisole.

**Table 2:** Effect of solvent on the cross-coupling reaction^a^.

Entry	Solvent	Time (min)	Yield^b^ (%)

1	95% EtOH	15	93
2	EtOH	20	89
3	CH_3_COCH_3_	240	85
4	CH_3_OH	25	82
5	H_2_O	90	50
6	DMF	300	45
7	CH_3_CN	420	88
8	DMSO	120	65

^a^Reaction conditions: 4-iodoanisole (1.0 mmol), phenylboronic acid (1.2 mmol), K_2_CO_3_ (2.0 mmol), Cell–OPPh_2_–Pd^0^ (0.015 g, 0.005 mmol of Pd), and 5.0 cm^3^ solvent heating under reflux in air. ^b^Isolated yield based on 4-iodoanisole.

**Table 3:** Effect of the amount of Cell–OPPh_2_–Pd^0^ catalyst on the cross-coupling reaction^a^.

Entry	Amount of catalyst (mmol Pd)	Time (min)	Yield^b^ (%)

1	0.15%	14	89
2	0.3%	10	91
3	0.5%	10	94
4	0.7%	10	93
5	0.9%	10	94

^a^Reaction conditions: 4-iodoanisole (1.0 mmol), phenylboronic acid (1.2 mmol), K_2_CO_3_ (2.0 mmol), Cell–OPPh_2_–Pd^0^, and 5.0 cm^3^ 95% ethanol heating under reflux in air. ^b^Isolated yield based on 4-iodoanisole.

We found that using K_2_CO_3_ as the base in 95% ethanol at 78 °C gave the coupled product, biphenyl **3e**, in 93% yield after 15 min (Table 1, entry 1). The other inorganic bases such as Na_2_CO_3_, Cs_2_CO_3_, NaOH, K_3_PO_4_·3H_2_O, CH_3_ONa, KOH and Na_3_PO_4_·12H_2_O were not as effective as K_2_CO_3_, and only afforded the coupling products in moderate to low yield (Table 1, entries 2–8). Among the screened bases, K_2_CO_3_ proved to be the best and was thus chosen as the base in the Suzuki–Miyaura reaction. From Table 2, we found that using 95% ethanol as the solvent gave the highest yield, i.e., 93% (Table 2, entries 1–8). The results in Table 3 showed that the loading of catalyst has an effect on the yield. When 0.15 mmol % of the catalyst was used, the yield of the product reached 89%. On increasing the amount of catalyst from 0.3 to 0.5 mmol %, the reaction yield rose from 91% to 94%. Further increasing the amount of catalyst had apparently no significant effect on the reaction yield. Therefore, 0.5 mmol % of the catalyst was enough to push the reaction to completion (Table 3, entry 3).

**Scheme 3:**
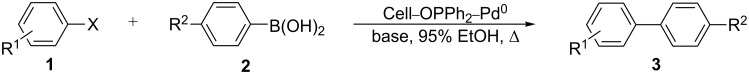
Reaction of aryl halides with arylboronic acids.

To examine the scope for this coupling reaction, a variety of substituted aryl halides were coupled with different arylboronic acids in 95% ethanol in the presence of a catalytic amount of Cell–OPPh_2_–Pd^0^ (0.5 mmol % Pd) with K_2_CO_3_ as base (Scheme 3). The typical experimental results are summarized in Table 4.

**Table 4:** The coupling reactions of aryl halides with various arylboronic acids^a^.

Entry	Aryl halide **1**	Arylboronic acid **2**	Time (min)	Yield^b^ (%)	Product **3**

1	C_6_H_5_I	C_6_H_5_B(OH)_2_	20	85	**3a**
2	4-NO_2_-C_6_H_4_I	C_6_H_5_B(OH)_2_	10	95	**3b**
3	4-Cl-C_6_H_4_I	C_6_H_5_B(OH)_2_	10	92	**3c**
4	4-Me-C_6_H_4_I	C_6_H_5_B(OH)_2_	15	85	**3d**
5	4-MeO-C_6_H_4_I	C_6_H_5_B(OH)_2_	15	93	**3e**
6	4-MeO-C_6_H_4_I	4-MeO-C_6_H_4_B(OH)_2_	18	65	**3f**
7	4-MeO-C_6_H_4_I	4-Me-C_6_H_4_B(OH)_2_	15	95	**3g**
8	4-MeO-C_6_H_4_I	4-Cl-C_6_H_4_B(OH)_2_	15	93	**3h**
9	4-MeO-C_6_H_4_I	4-F-C_6_H_4_B(OH)_2_	15	94	**3i**
10	4-MeO-C_6_H_4_I	4-CHO-C_6_H_4_B(OH)_2_	15	96	**3j**
11	4-NO_2_-C_6_H_4_I	4-MeO-C_6_H_4_B(OH)_2_	15	98	**3k**
12	4-NO_2_-C_6_H_4_I	4-Me-C_6_H_4_B(OH)_2_	8	68	**3l**
13	4-NO_2_C_6_H_4_I	4-Cl-C_6_H_4_B(OH)_2_	15	86	**3m**
14	4-NO_2_-C_6_H_4_I	4-F-C_6_H_4_B(OH)_2_	15	96	**3n**
15	4-NO_2_-C_6_H_4_I	4-CHO-C_6_H_4_B(OH)_2_	10	91	**3o**
16	C_6_H_5_Br	C_6_H_5_B(OH)_2_	15	63	**3a**
17	4-Me-C_6_H_4_Br	C_6_H_5_B(OH)_2_	30	60	**3d**
18	4-MeO-C_6_H_4_Br	C_6_H_5_B(OH)_2_	25	75	**3e**
19	4-NO_2_-C_6_H_4_Br	C_6_H_5_B(OH)_2_	15	96	**3b**
20	3-NO_2_-C_6_H_4_Br	C_6_H_5_B(OH)_2_	10	93	**3p**
21	4-Br-C_6_H_5_Br	C_6_H_5_B(OH)_2_	50	48	**3q**
22	4-MeO_2_C-C_6_H_4_Br	C_6_H_5_B(OH)_2_	20	98	**3r**
23	4-NO_2_-C_6_H_4_Cl	C_6_H_5_B(OH)_2_	12 h	23	**3b**

^a^Reaction conditions: aryl halide (1.0 mmol), arylboronic acid (1.2 mmol), K_2_CO_3_ (2.0 mmol), Cell–OPPh_2_–Pd^0^ (0.015 g, 0.005 mmol of Pd), and 5.0 cm^3^ 95% ethanol heating under reflux in air. ^b^Isolated yield based on aryl halide.

The data presented in Table 4 show that the Cell–OPPh_2_–Pd^0^ catalyst was highly effective for both aryl bromides and aryl iodides. Most of these reactions proceeded rapidly and were complete within 20 min. The catalytic performance was excellent for substrates with electron-withdrawing groups (Table 4, entries 2, 3, 19, 20 and 22) and was only slightly lower for substrates with electron-donating groups (Table 4, entries 4 and 17), except for 4-iodoanisole and 4-bromoanisole (Table 4, entries 5 and 18). As expected, the reactivity of aryl bromides was slightly lower than that of the corresponding aryl iodides and in these cases a prolonged time was required. The reaction of aryl chlorides with arylboronic acids was sluggish and gave only small amounts of products in acceptable times.

**Figure 5:**
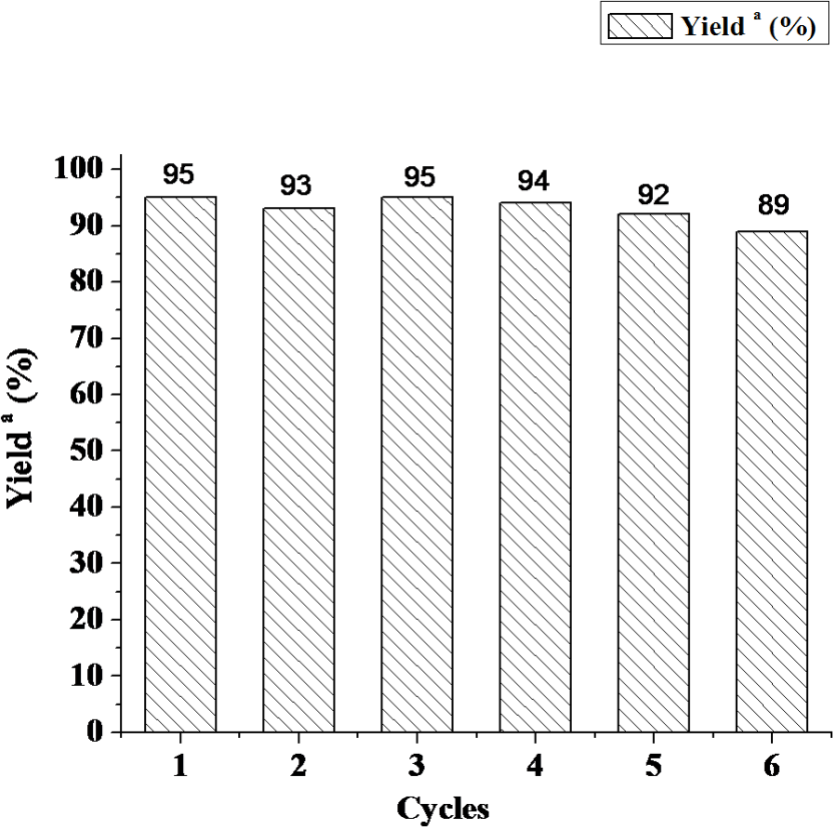
Recycling of Cell–OPPh_2_–Pd^0^ for the Suzuki reaction. Reaction conditions: 4-iodoanisole (1.0 mmol), phenylboronic acid (1.2 mmol), K_2_CO_3_ (2.0 mmol), Cell–OPPh_2_–Pd^0^ (0.015 g, 0.005 mmol of Pd), and 5.0 cm^3^ 95% ethanol heating under reflux in air. ^a^Isolated yield based on 4-iodoanisole.

The reusability of the supported catalyst is a very important theme from the standpoint of green chemistry and for its suitability for commercial applications. Finally, we explored the reusability of the Cell–OPPh_2_–Pd^0^ catalyst again with the reaction of 4-iodoanisole with phenylboronic acid as the model reaction. After the first run, the catalyst was filtered and extensively washed with ethanol and dried in vacuo. Then the catalyst was reused directly under the same conditions mentioned above. The results are shown in Figure 5.

It can be seen from the results that the catalyst could be reused up to six times whilst still retaining good catalytic activity. Characterization of the reused catalysts by TEM showed that the slight agglomeration of the palladium nanoparticles had no apparently effect on its catalytic performance (Figure 4).

## Conclusion

Nanopalladium immobilized on the surface of Cell–OPPh_2_ has high catalytic activity in Suzuki–Miyaura cross-coupling reactions in 95% aqueous ethanol under atmospheric conditions. The catalyst can be easily separated and recovered from the reaction mixture by filtration and reused up to six times without any noticeable loss of activity. This simple procedure, coupled with the easy recovery and reusability of the catalyst is expected to contribute to the development of chemical processes and products.

## Experimental

### General

Melting points were measured on an Electrothermal X6 microscopic digital melting point apparatus. IR spectra were recorded on a Bruker Equinox-55 spectrometer as KBr pellets. ^1^H NMR spectra were obtained with a 300 MHz Bruker Avance instrument with CDCl_3_ as solvent and TMS as internal standard. Elemental analyses were performed on a Perkin-Elmer EA2400II elemental analyzer. The elemental palladium content of the polymeric catalysts was determined by Perkin-Elmer Optima 2000DV inductively coupled plasma (ICP) spectroscopy. Scanning electron microscopy (SEM) was performed with a Philips XL 30ESEM instrument. Transmission electron microscopy (TEM) was performed with a Philips Tecnai instrument operating at 40–100 kV. The chemicals were obtained from commercial sources and used as received.

#### Preparation of diphenylphosphinite cellulose (Cell–OPPh_2_)

A mixture of cellulose (3.0 g) and dry pyridine (120 cm^3^) in a round-bottomed flask was vigorous stirred at 80–90 °C for 30 min. After being cooled to room temperature, diphenylchlorophosphine (12 cm^3^) was added to the mixture and the reaction mixture stirred at room temperature for 5 d. The reaction mixture was filtered and the solid obtained washed with a large volume of ethanol and dried under vacuum at 60 °C to give white Cell–OPPh_2_.

#### Preparation of Cell–OPPh_2_–Pd^0^ complex

Cell–OPPh_2_ (2.0 g) was added to a solution of PdCl_2_ (0.16 g, 0.9 mmol) in 95% ethanol (30 cm^3^). The mixture was heated under reflux for 24 h, allowed to cool and filtered. The resulting product was washed successively with ethanol (3 × 25 cm^3^) and Et_2_O (3 × 25 cm^3^) and dried under vacuum at 60 °C to give the dark gray polymeric palladium(0) complex (Cell–OPPh_2_–Pd^0^).

#### General procedure for the Suzuki–Miyaura cross-coupling reaction

In a typical experiment, the Cell–OPPh_2_–Pd^0^ catalyst (0.005 mmol of Pd) was added to a mixture of aryl halide (1.0 mmol), arylboronic acid (1.2 mmol), and K_2_CO_3_ (2.0 mmol) in 95% ethanol (5 cm^3^), and the reaction mixture was stirred and heated under reflux. After the reaction was judged to be complete by TLC analysis, the catalyst was removed by filtration, washed with ethanol (3 × 4 cm^3^) and dried under vacuum for the next run. The organic fractions were then concentrated on a rotary evaporator to obtain the desired biaryl in excellent yield. The crude products were further purified by recrystallization. All of the products (**3a**–**3r)** are known compounds and their data was identical to that reported in literature. The data of some products are as follows:

**3b:** Mp 113–114 °C. ^1^H NMR (CDCl_3_, 300 MHz) δ 8.30 (d, *J* = 8.7 Hz, 2H, Ar-H), 7.74 (d, *J* = 8.7 Hz, 2H, Ar-H), 7.62 (t, *J* = 7.5 Hz, 2H, Ar-H), 7.50–7.45 (m, 3H, Ar-H); IR (KBr) ν 3076, 1595, 1541, 1347, 1105, 854, 741, 700 cm^−1^.

**3e:** Mp 86–87 °C. ^1^H NMR (CDCl_3_, 300 MHz) δ 7.56 (d, *J* = 7.3 Hz, 4H, Ar-H), 7.44 (d, *J* = 7.8 Hz, 2H, Ar-H), 7.30 (d, *J* = 7.2 Hz, 1H, Ar-H), 6.99 (d, *J* = 6.7 Hz, 2H, Ar-H), 3.85 (s, 3H, CH_3_O); IR (KBr) ν 2961, 1606, 1522, 1488, 1251, 1201, 1036, 833, 760, 699 cm^−1^.

**3k:** Mp 105–107 °C. ^1^H NMR (CDCl_3_, 300 MHz) δ 8.23 (d, *J* = 8.9 Hz, 2H, Ar-H), 7.67 (d, *J* = 7.8 Hz, 2H, Ar-H), 7.56 (d, *J* = 7.2 Hz, 2H, Ar-H), 7.00 (d, *J* = 6.7 Hz, 2H, Ar-H), 3.86 (s, 3H, CH_3_O); IR (KBr) ν 2930, 2835, 1593, 1508, 1344, 1252, 1187, 1108, 1016, 857, 757, 697 cm^−1^.

**3n:** Mp 122–125 °C. ^1^H NMR (CDCl_3_, 300 MHz) δ 8.29 (d, *J* = 8.3 Hz, 2H, Ar-H), 7.69 (d, *J* = 8.8 Hz, 2H, Ar-H), 7.62–7.60 (m, 2H), 7.21–7.19 (m, 2H); IR (KBr) ν 3075, 1599, 1518, 1348, 1233, 1113, 853, 756, 728 cm^−1^.

## Supporting Information

File 1 IR for catalyst and selected products.

File 2 ^1^H NMR spectra for selected products.
