# Supplementary material for: Air-stable, recyclable, and time-efficient diphenylphosphinite cellulose-supported palladium nanoparticles as a catalyst for Suzuki–Miyaura reactions
Source: Beilstein J Org Chem. 2011 Mar 30;7:378–85. doi: 10.3762/bjoc.7.48 (PMC3079107; doi:10.3762/bjoc.7.48)
Supplement: File 1 — IR for catalyst and selected products. [file Beilstein_J_Org_Chem-07-378-s001.pdf]

Supporting Information

for

Air-stable, recyclable, and time-efficient diphenylphosphinite cellulose-supported palladium nanoparticles as a catalyst for Suzuki–Miyaura reactions

Qingwei Du and Yiqun Li*

Address: Department of Chemistry, Jinan University, 510632 Guangzhou, China

Email: Yiqun Li* - tlyq@jnu.edu.cn

*Corresponding author

IR for catalyst and selected products

Content

IR Cell–$OPPh_2$. S2
IR Cell–$OPPh_2$–Pd^0.......... S2
IR **3b**. S3
IR **3e**. S3
IR **3k**. S4
IR **3n**. S4

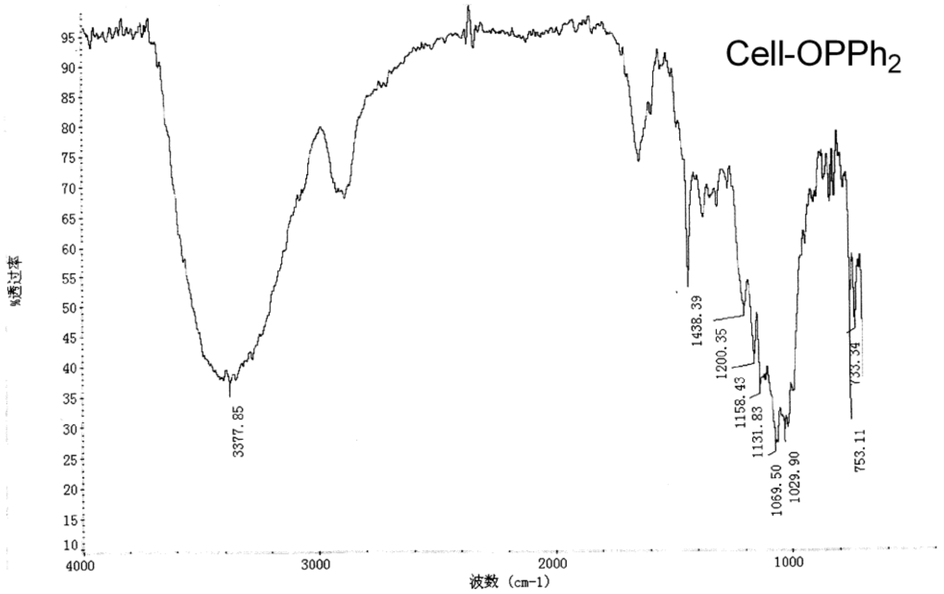

Figure 1: IR of Cell–$OPPh_2$.

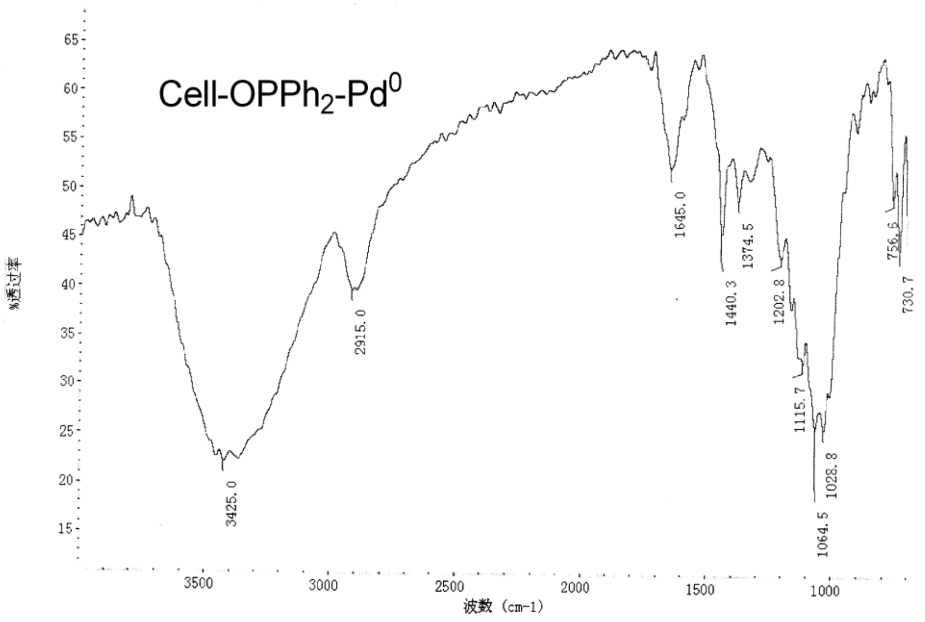

Figure 2: IR of Cell–$OPPh_2$–Pd^0.

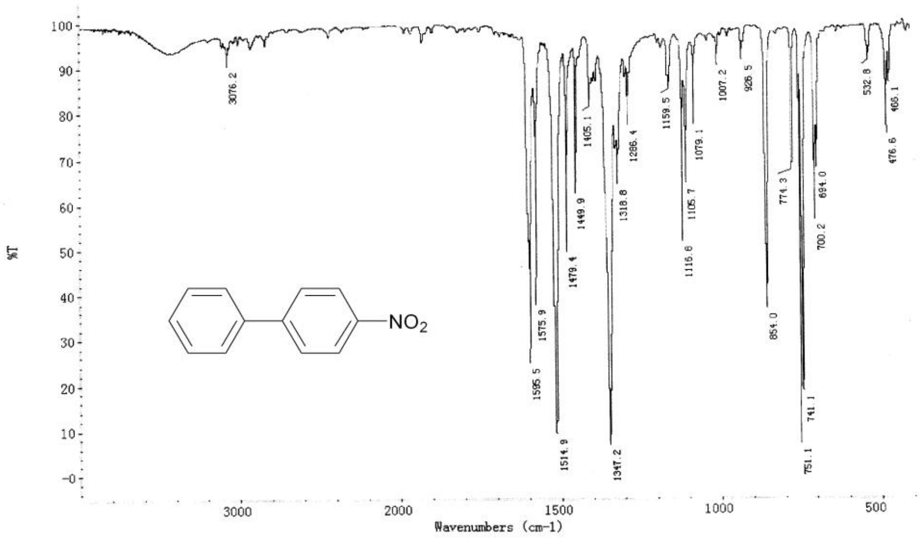

Figure 3: IR of 4-nitro-biphenyl (**3b**).

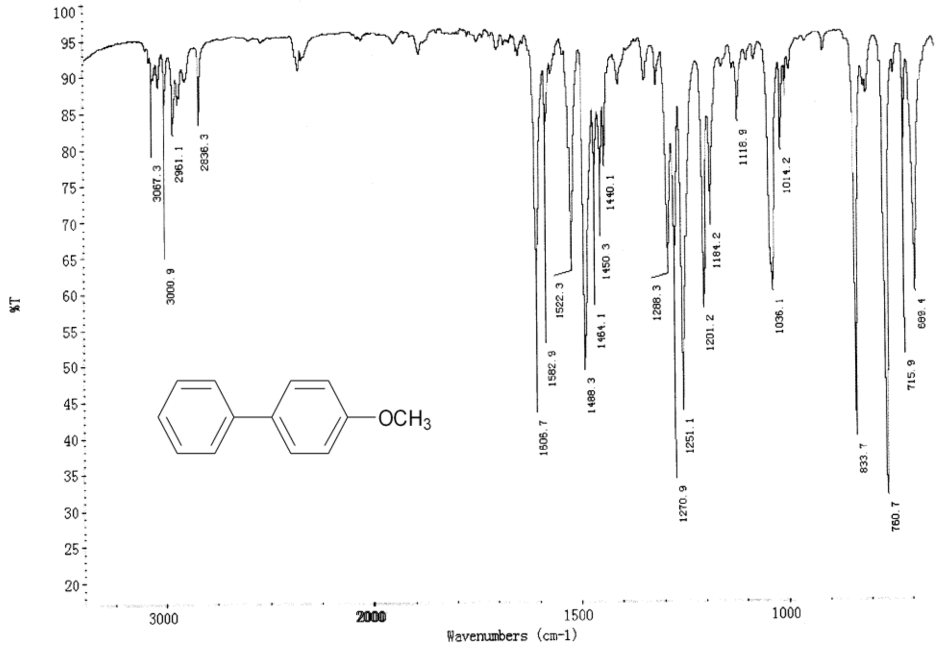

Figure 4: IR of 4-methoxy-biphenyl (**3e**).

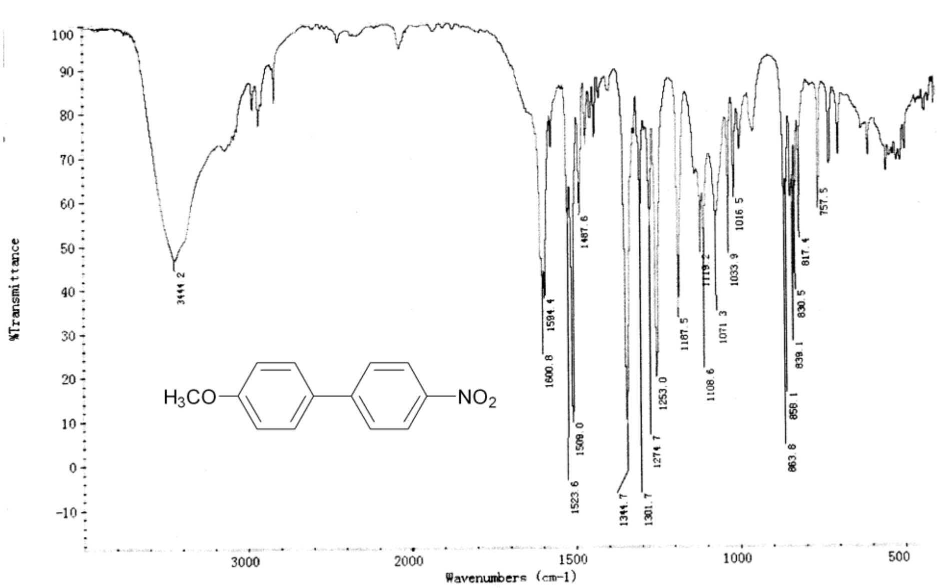

Figure 5: IR of 4'-methoxy-4-nitrobiphenyl (**3k**).

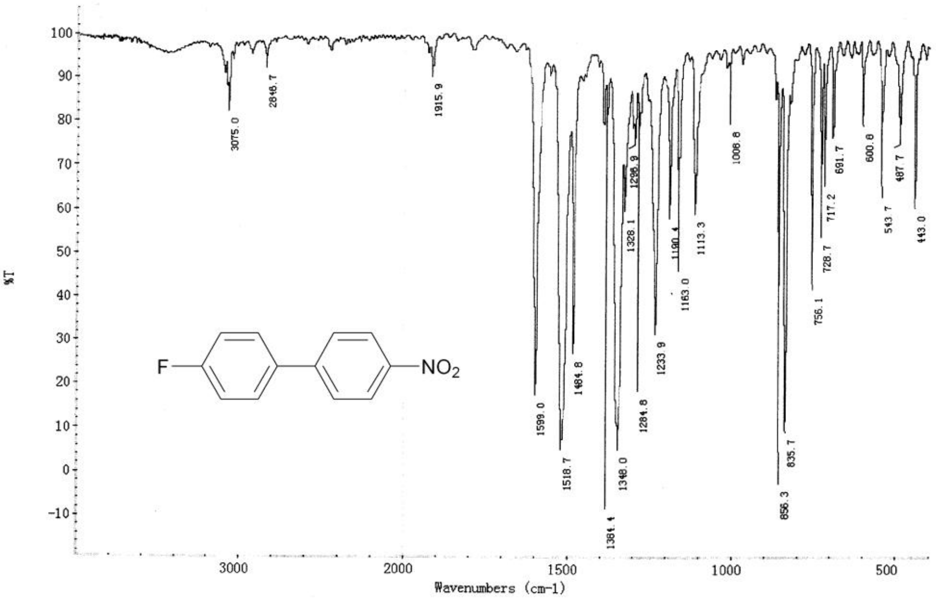

Figure 6: IR of 4-fluoro-4'-nitrobiphenyl (**3n**).
