# Supplementary material for: Air-stable, recyclable, and time-efficient diphenylphosphinite cellulose-supported palladium nanoparticles as a catalyst for Suzuki–Miyaura reactions
Source: Beilstein J Org Chem. 2011 Mar 30;7:378–85. doi: 10.3762/bjoc.7.48 (PMC3079107; doi:10.3762/bjoc.7.48)
Supplement: File 2 — 1H NMR spectra for selected products. [file Beilstein_J_Org_Chem-07-378-s002.pdf]

Supporting Information

for

Air-stable, recyclable, and time-efficient diphenylphosphinite cellulose-supported palladium nanoparticles as a catalyst for Suzuki–Miyaura reactions

Qingwei Du and Yiqun Li*

Address: Department of Chemistry, Jinan University, 510632 Guangzhou, China

Email: Yiqun Li* - tlyq@jnu.edu.cn

*Corresponding author

^{1}H NMR for selected products

Content

^{1}H NMR **3b**.. S2
^{1}H NMR **3e**.. S2
^{1}H NMR **3k**.. S3
^{1}H NMR **3n**.. S3

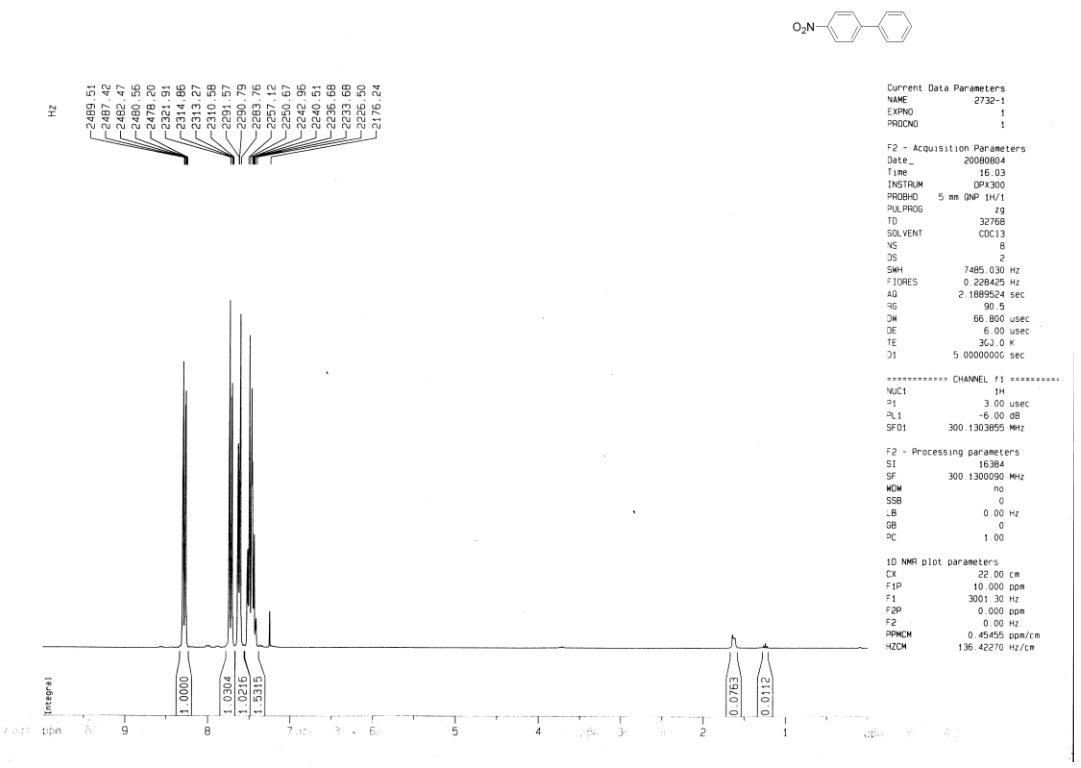

Figure 1: ^{1}H NMR of 4-nitrobiphenyl (**3b**).

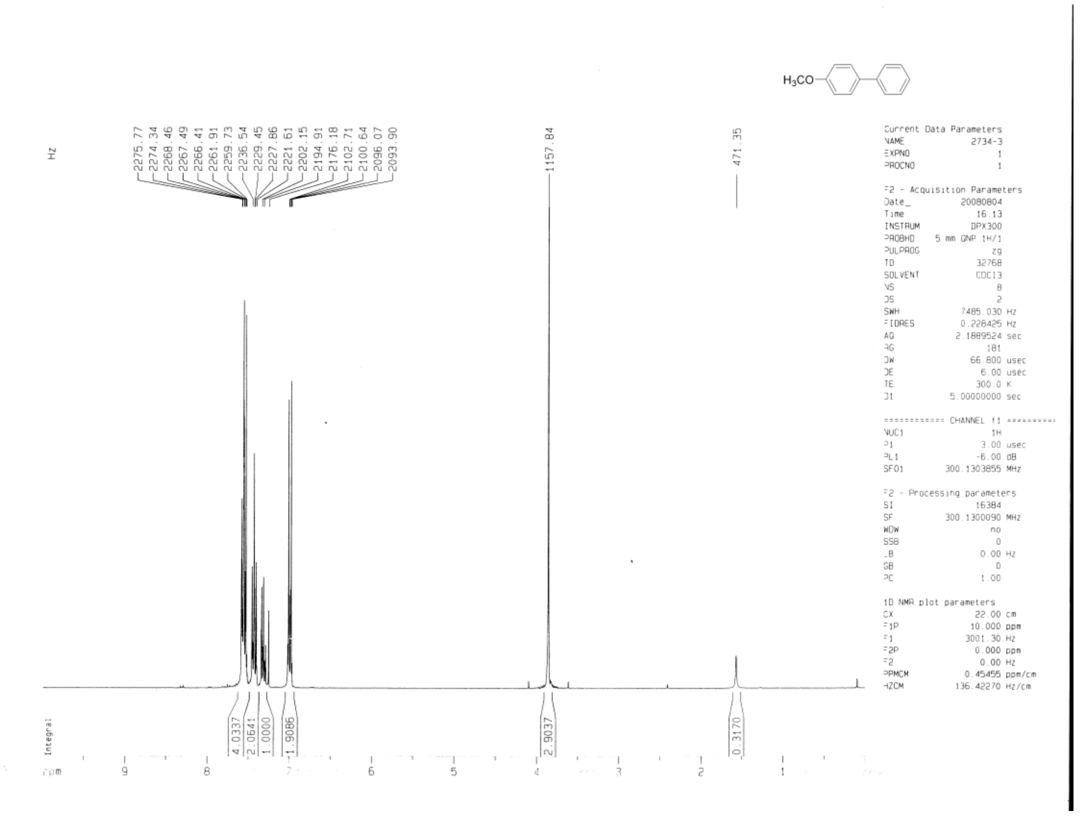

Figure 2: ^{1}H NMR of 4-methoxybiphenyl (**3e**).

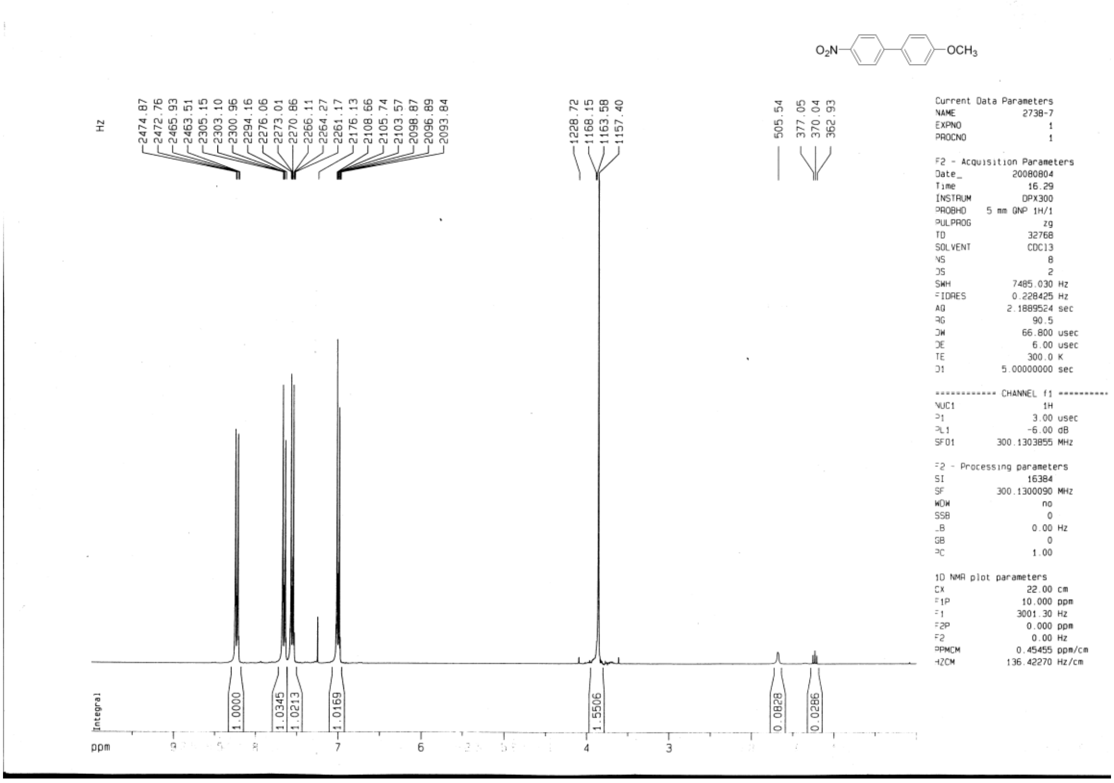

Figure 3: ^{1}H NMR of 4'-methoxy-4-nitrobiphenyl (**3k**).

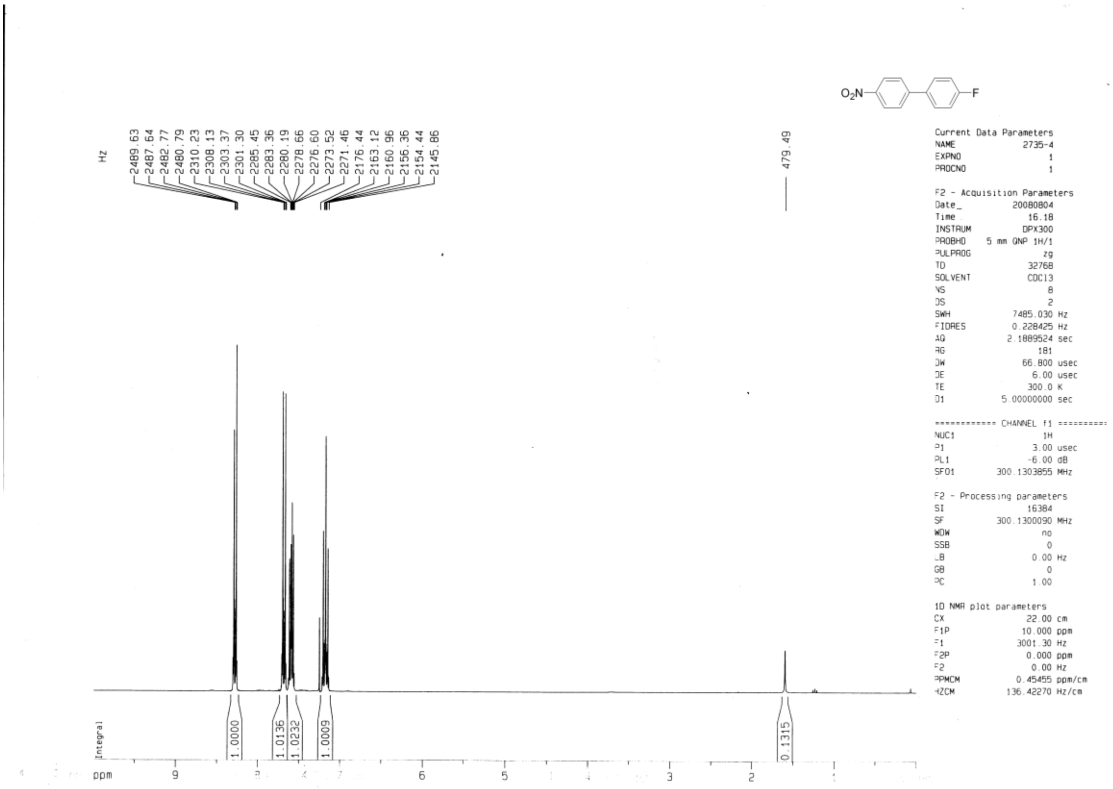

Figure 4: ^{1}H NMR of 4-fluoro-4'-nitrobiphenyl (**3n**).
